# A personalized, multiomics approach identifies genes involved in cardiac hypertrophy and heart failure

**DOI:** 10.1038/s41540-018-0046-3

**Published:** 2018-02-24

**Authors:** Marc Santolini, Milagros C. Romay, Clara L. Yukhtman, Christoph D. Rau, Shuxun Ren, Jeffrey J. Saucerman, Jessica J. Wang, James N. Weiss, Yibin Wang, Aldons J. Lusis, Alain Karma

**Affiliations:** 1 Center for Interdisciplinary Research on Complex Systems, Department of Physics, Northeastern University, Boston, MA USA; 2 Center for Complex Network Research, Department of Physics, Northeastern University, Boston, MA USA; 3 Center for Cancer Systems Biology (CCSB) and Department of Cancer Biology, Dana-Farber Cancer Institute, 450 Brookline Ave., Boston, MA 02215 USA; 4 Department of Medicine, Brigham and Women’s Hospital, Harvard Medical School, 75 Francis Street, Boston, MA 02115 USA; 5 Department of Microbiology, Immunology and Molecular Genetics, University of California, Los Angeles, CA 90095 USA; 6 Department of Molecular, Cell, and Developmental Biology, David Geffen School of Medicine, University of California, Los Angeles, Los Angeles, CA USA; 7 Departments of Anesthesiology, Physiology and Medicine, Cardiovascular Research Laboratories, David Geffen School of Medicine, University of California, Los Angeles, CA 90095 USA; 8 Department of Biomedical Engineering, University of Virginia, Charlottesville, VA 22908 USA; 9 Departments of Medicine and Human Genetics, David Geffen School of Medicine, David Geffen School of Medicine, University of California, Los Angeles, CA 90095 USA

## Abstract

A traditional approach to investigate the genetic basis of complex diseases is to identify genes with a global change in expression between diseased and healthy individuals. However, population heterogeneity may undermine the effort to uncover genes with significant but individual contribution to the spectrum of disease phenotypes within a population. Here we investigate individual changes of gene expression when inducing hypertrophy and heart failure in 100 + strains of genetically distinct mice from the Hybrid Mouse Diversity Panel (HMDP). We find that genes whose expression fold-change correlates in a statistically significant way with the severity of the disease are either up or down-regulated across strains, and therefore missed by a traditional population-wide analysis of differential gene expression. Furthermore, those “fold-change” genes are enriched in human cardiac disease genes and form a dense co-regulated module strongly interacting with the cardiac hypertrophic signaling network in the human interactome. We validate our approach by showing that the knockdown of *Hes1*, predicted as a strong candidate, induces a dramatic reduction of hypertrophy by 80–90% in neonatal rat ventricular myocytes. Our results demonstrate that individualized approaches are crucial to identify genes underlying complex diseases as well as to develop personalized therapies.

## Personalized medicine: uncovering missed disease genes

A multitude of genes associated with complex diseases are revealed by a novel personalized, as opposed to population-level, analysis of differential gene expression. While traditional investigations of the genetic basis of complex diseases assume homogeneity across individuals and identify genes differentially expressed between a diseased and a healthy population, Northeastern University and University of California Los Angeles researchers have identified a different class of disease genes that exhibit heterogeneous up and down-regulation across 100 genetically distinct mouse strains subject to a stressor inducing heart failure, but show no significant change of expression at the population level. The results, validated by in vitro knockdown, demonstrate that individualized approaches are crucial to unmask all genes involved in complex diseases, opening new avenues for the development of personalized therapies.

## Introduction

Contrary to “Mendelian” diseases where causality can be traced back to strong effects of a single gene, common diseases result from modest effects of many interacting genes.^1^ Understanding which genes are involved and how they affect diseases is a major challenge for designing appropriate therapies.

Heart failure (HF) is a well-studied example of a genetically complex disease involving multiple processes that eventually lead to a common phenotype of abnormal ventricular function and cardiac hypertrophy.^2^ Numerous studies have attempted to pinpoint differentially expressed genes (DEGs) to find biomarkers for the prognosis of the disease and the design of appropriate drugs,^3^ as well as explore underlying affected signaling pathways.^4^ Such studies typically compare the average gene expression between samples in healthy and diseased states, such as non-failing vs failing hearts in murine,^5^ canine,^6^ or human samples (see^7^ for a broad review). Genes are ranked by the strength of their differential expression, and top ranking genes are further investigated for pathway enrichment and biomarker potential. However, because of the different genetic backgrounds of the surveyed individuals, as well as different severities of HF, those studies show very limited overlap of DEGs. While separate studies typically identify tens to hundreds of DEGs, not a single DEG is common to all studies.^7^ Moreover, it is unclear whether the healthy state is itself a well-defined unique state. In particular, several studies have shown that, due to compensatory mechanisms involved in homeostasis, different combinations of ion channel conductances in neurons and cardiac cells can lead to a normal electrophysiological phenotype, e.g., a similar bursting pattern of motor neurons or a similar cardiac action potential and calcium transient.^8,9^ This has led to the concept that genetically distinct individuals represent different “Good Enough Solutions” corresponding to distinct gene expression patterns underlying a healthy phenotype. Different combinations of gene expression in a healthy state resulting from genetic variations would be expected to yield different DEGs in a diseased state. Thus, small numbers of DEGs that are only shared by a subset of individuals, and would be missed by a standard population-wide DEG analysis, could in principle have a causal role. Identifying these genes remains a central challenge in personalized medicine.^10,11^

In order to explore the variability of individual trajectories leading to hypertrophy and HF, we leverage the Hybrid Mouse Diversity Panel (HMDP), a model system consisting of >100 genetically diverse strains of mice that we described previously^12,13^ (see Methods). Gene expression and phenotypic data are acquired before and 3 weeks after implantation of a pump delivering isoproterenol (ISO). This pathological stressor induces a global response characterized mainly by cardiac hypertrophy along with more marginal changes in chamber dilation and contractile function at the population level.^12^ As a result, we primarily focus on the identification of genes relevant for cardiac hypertrophy. Expression data is collected at the whole heart level and the Total Heart Weight is used to quantify the degree of cardiac hypertrophy. Importantly, the severity of the hypertrophic response is highly variable among strains, ranging from almost no hypertrophy to up to an 80% increase of heart mass. Our study is directed at understanding why certain individuals are more susceptible to or protected against cardiac hypertrophy due to their genetic backgrounds. Because mice from the same strains are isogenic and renewable, the HMDP offers the possibility to analyze differential gene expression and phenotype change in a unique setting where subjects in the control population can be matched to a subject with the same genetic background in the treated population. In that setup, one can correlate the stressor-related gene expression change with the corresponding phenotype change (in our case, heart mass increase) while controlling for genetic background, thereby disentangling intra-strain (stressor-induced) and inter-strain (genetics-induced) variations. In the specific case of HF onto which we focus here, such data could not be obtained in human studies where heart tissue biopsies are extracted from either healthy donor hearts or explanted hearts of late stage HF patients in a genetically diverse population.^14^ One would indeed require a population of identical twins in which one twin for each pair of twins is a heart donor and the other twin is a late stage HF patient. As such, gene expression data obtained from those biopsies can only be used to perform a population-level differential gene expression analysis. In contrast, here we identify relevant genes by correlating strain-specific temporal changes of gene expression, i.e., differential expression between a post-ISO mouse and another pre-ISO mouse from the same strain, with the corresponding strain-specific changes of phenotype, i.e., ratio of heart mass between the post- and pre-ISO mice of the same strain.

Concretely (see Methods), we calculate the Pearson coefficient of correlation $C_j$ between the strain-specific fold-change of expression of gene $j$ among $N$ different strains

1 $${\boldsymbol{F}}_{\boldsymbol{j}} = \left( {{\mathbf{log}}_2\frac{{{\boldsymbol{E}}_1^\prime \left( {\boldsymbol{j}} \right)}}{{{\boldsymbol{E}}_1\left( {\boldsymbol{j}} \right)}},{\mathbf{log}}_2\frac{{{\boldsymbol{E}}_2^\prime \left( {\boldsymbol{j}} \right)}}{{{\boldsymbol{E}}_2\left( {\boldsymbol{j}} \right)}}, \ldots ,{\mathbf{log}}_2\frac{{{\boldsymbol{E}}_{\boldsymbol{N}}^\prime \left( {\boldsymbol{j}} \right)}}{{{\boldsymbol{E}}_{\boldsymbol{N}}\left( {\boldsymbol{j}} \right)}}} \right)$$

where $E_i\left( j \right)$ and $E_i^\prime \left( j \right)$ are the expression levels of gene *j* for two isogenic mice of the $i{\rm th}$ strain before and after ISO treatment, respectively, and the strain-specific fold-change of heart mass among different strains

2 $${\boldsymbol{F}}_{\boldsymbol{m}} = \left( {{\mathbf{log}}_2\frac{{{\boldsymbol{m}}_1^\prime }}{{{\boldsymbol{m}}_1}},{\mathbf{log}}_2\frac{{{\boldsymbol{m}}_2^\prime }}{{{\boldsymbol{m}}_2}}, \ldots ,{\mathbf{log}}_2\frac{{{\boldsymbol{m}}_{\boldsymbol{N}}^\prime }}{{{\boldsymbol{m}}_{\boldsymbol{N}}}}} \right)$$

where $m_i$ and $m_i^\prime$ are the total heart mass of isogenic mice of the $i{\rm th}$ strain before and after ISO treatment, respectively; we use log_2_ of expression fold-change to normalize microarrary data and log_2_ of heart mass fold-change for consistency (Methods). This correlation method of differential gene expression analysis identifies a set of DEGs, referred to hereafter as “fold-change” (FC) genes, for which the absolute value of $C_j$ is above a threshold of statistical significance determined by randomization of the data as detailed further in the article and the Methods. The ability to study a large number ($N\sim 100$) of strains using the HMDP is essential to have enough statistical power to establish such a correlation, a power that has been lacking from previous studies limited to small numbers of strains.^15–18^ Moreover, the correlation coefficients $C_j$ cannot be calculated in the setting of traditional clinical studies since the fold change of gene expression or heart mass of subjects with different genetic background is meaningless. Conversely, it is possible to analyze the HMDP data set using the same type of population-level differential gene expression analysis used in clinical studies, such as SAM (Significance Analysis of Microarrays).^19^ Applied to the HMDP data set, a method like SAM identifies a gene *j* as differentially expressed if the expression data in the control population $\left( {{\mathrm{log}}_2E_1\left( j \right),\log _2E_2\left( j \right),\, \ldots \,,\,\log _2E_N\left( j \right)} \right)$ and the treated population $\left( {\log _2E_1^\prime \left( j \right),\log _2E_2^\prime \left( j \right),\, \ldots \,,\,{\mathrm{log}}_2E_N^\prime \left( j \right)} \right)$ have statistically distinguishable mean values, irrespective of the individual reaction to the stressor $F_m$. As further detailed in the methods, SAM genes do not consider the strength of phenotypic change $F_m$ but rely on the average gene expression change $\langle {F_j} \rangle$, while FC genes consider both expression and phenotypic changes through an interaction term $\langle {F_jF_m} \rangle.$

Based on our computation of the $C_j$ correlation coefficients, we find a small set of 36 FC genes and compare them to a larger set of genes identified with SAM (referred to hereafter as SAM genes). Interestingly, the sets of FC and SAM genes have negligible overlap. The FC genes are not identified as significantly changed at the population level because they typically have opposite fold changes in low and high hypertrophy strains that cancel each other when averaged over all strains in the population-wide case. We show that the FC genes are strongly enriched in cardiac disease genes from previous Genome-Wide Association Studies (GWAS), while SAM genes are in contrary enriched in fibrosis genes. We then show that those two sets form two distinct communities in the co-expression network among healthy as well as ISO-injected strains and we identify potential transcription factors (TFs) to explain the observed co-regulation of FC genes. Moreover, we find that the proteins encoded by the FC genes, but not the SAM genes, interact predominantly with proteins belonging to a cardiac hypertrophic signaling network (CHSN) that has been shown to provide a predictive model of hypertrophy in relation to multiple stressors including ISO.^20^ Interestingly, we find that one of the FC genes, namely *Hes1*, is also a predicted TF and an important interactor with the CHSN. Using a knockdown approach, we find that it plays a major role in cardiac hypertrophy, allowing us to validate our personalized, multiomics approach.

## Results

### Two types of responses to stressor-induced cardiac hypertrophy and heart failure

**Fig. 1:**
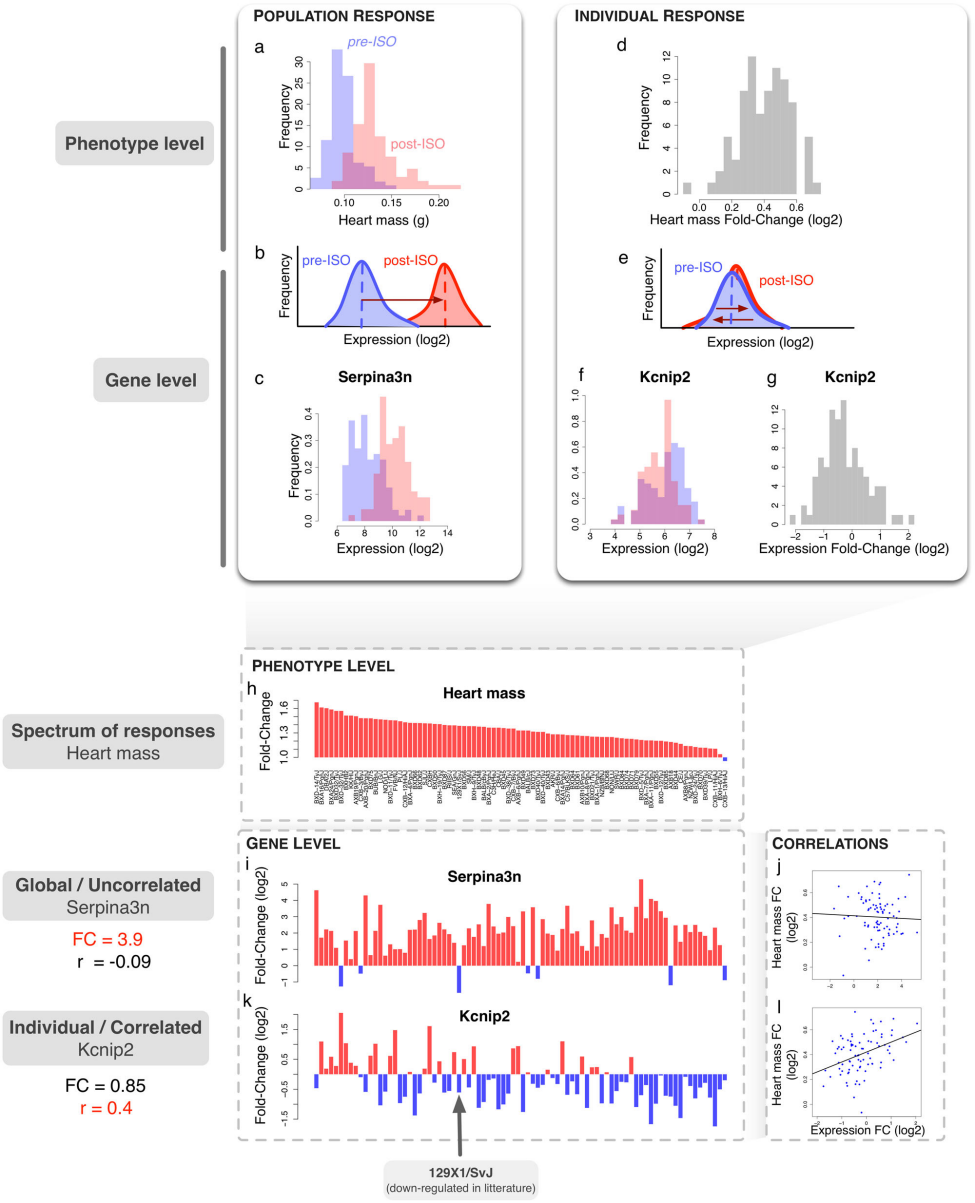
Two types of responses to stressor-induced heart failure. **a** Histograms of the pre-ISO (blue) and post-ISO (red) heart masses of the HMDP strains. **b** Typical Differentially Expressed Genes (DEGs) show clear population-average fold-change allowing distinguishing the two populations of strains. **c** An example of such strong DEG, namely *Serpina*3n. **d** Histogram of the heart mass fold-change (FC) computed for each strain from the HMDP. **e** Expression FC at the individual level can lead to cases were the population-average FC is null while the individual FCs are not. **f** *Kcnip2* is a good example of a gene with no population-wide average FC. **g** However, at the individual level, *Kcnip2* shows strong variations, as seen in the histogram of individual FCs at the strain level (log2 of post over pre-ISO expression ratio). In particular, some strains have a 4-fold decrease of expression (−2 in log2), while others have a 4-fold increase (+2). **h** For better visualization, the strain-specific heart mass FC is shown by decreasing strength. Red bars indicate increase and blue bars decrease in value. **i** *Serpina*3n log FC is shown with the same strain ordering than in (**h**). Its population-wide FC is high (3.9), with most strains showing a strong positive FC (red bars). **j** However, the correlation of *Serpina*3n FC with the heart mass FC is not significant (*r* = −0.09, *p* = 0.43). **k** On the other hand, *Kcnip2* shows a weak population-wide FC (FC = 0.85). In particular, some strains show an increased expression (red bars) while others show a decreased expression (blue bars). The red arrow indicates the 129 × 1/SvJ strain in which *Kcnip2* has previously been shown to be down-regulated during cardiac hypertrophy.^24^ **l** Contrary to *Serpina*3n, *Kcnip2* FC is significantly correlated to heart mass FC (*r* = 0.4, *p* = 1.5e-4), with increased expression corresponding to high hypertrophy and decreased expression corresponding to low hypertrophy

We begin with an example showing two distinct ways to describe the response to ISO in the HMDP (see Fig. 1 and Methods). First, one can note that ISO induces a global response across all strains, resulting in cardiac hypertrophy. This is seen in Fig. 1a, where the distribution of heart mass among the post-ISO strains can clearly be distinguished from the pre-ISO distribution (*p* < 2.2e-16 under Student *t*-test). At the gene level, such a response is typically analyzed by looking for DEGs at the population level, i.e., genes for which the change in average expression with the stressor is significantly greater than the variability with and without the stressor (Fig. 1b). Typical tools include *t*-test,^21^ SAM,^19^ or LIMMA.^22^ Genes found with these methods have a differential expression profile at the population level and are therefore potential biomarkers of the trait of interest (see microarray data for *Serpina3n*, an example high-ranking SAM gene, in Fig. 1c). However, despite the global response in the level of gene expression to ISO, the degree of hypertrophy among individual strains is highly variable, from almost none to an 80% increase of heart weight (Fig. 1d). This calls for an evaluation of the strength of differential gene expression at the individual level. In particular, a whole new class of genes becomes available for analysis. Indeed, even if a gene does not show population-wide average differential expression, it can show extensive variation at the individual, strain-specific level (Fig. 1e). This is the case for the gene *Kcnip2* encoding the protein KChIP2, which interacts with pore forming subunits (Kv4.2 and Kv4.3) of the transient outward current *I*_to_ expressed in heart, and which has been implicated in cardiac hypertrophy.^23–25^ Though not showing population-wide differential expression (Fig. 1f), its individual fold-change of expression can vary drastically from 2-fold decrease to a 2-fold increase depending on the considered strain (Fig. 1g). Interestingly, when comparing the individual variations of those two types of genes with the degree of hypertrophy (Fig. 1h, i, k), one can see that global DEGs are not necessarily good descriptors of the individual changes of phenotype (Fig. 1j), unlike the second type of genes missed by a traditional population-wide method (Fig. 1l). In particular, in the case of *Kcnip2*, we observe a significant positive correlation with the severity of hypertrophy (*r* = 0.4, *p* = 1.5e-4). This is particularly interesting since *Kcnip2* has previously been shown to be down-regulated during cardiac hypertrophy^24,26^ in the strain 129 × 1/SvJ. While we confirm this finding, we also observe that it is unusual in a broader context, and that *Kcnip2* is most of the time up-regulated in strains with marked hypertrophy.

In the following, we generalize these observations to identify a larger set of genes that, like *Kcnip2*, have an individual FC correlated to the severity of hypertrophy, and we compare this set to the complete set of DEGs identified by the population-level SAM method.

### Identification of genes associated with the severity of hypertrophy

**Fig. 2:**
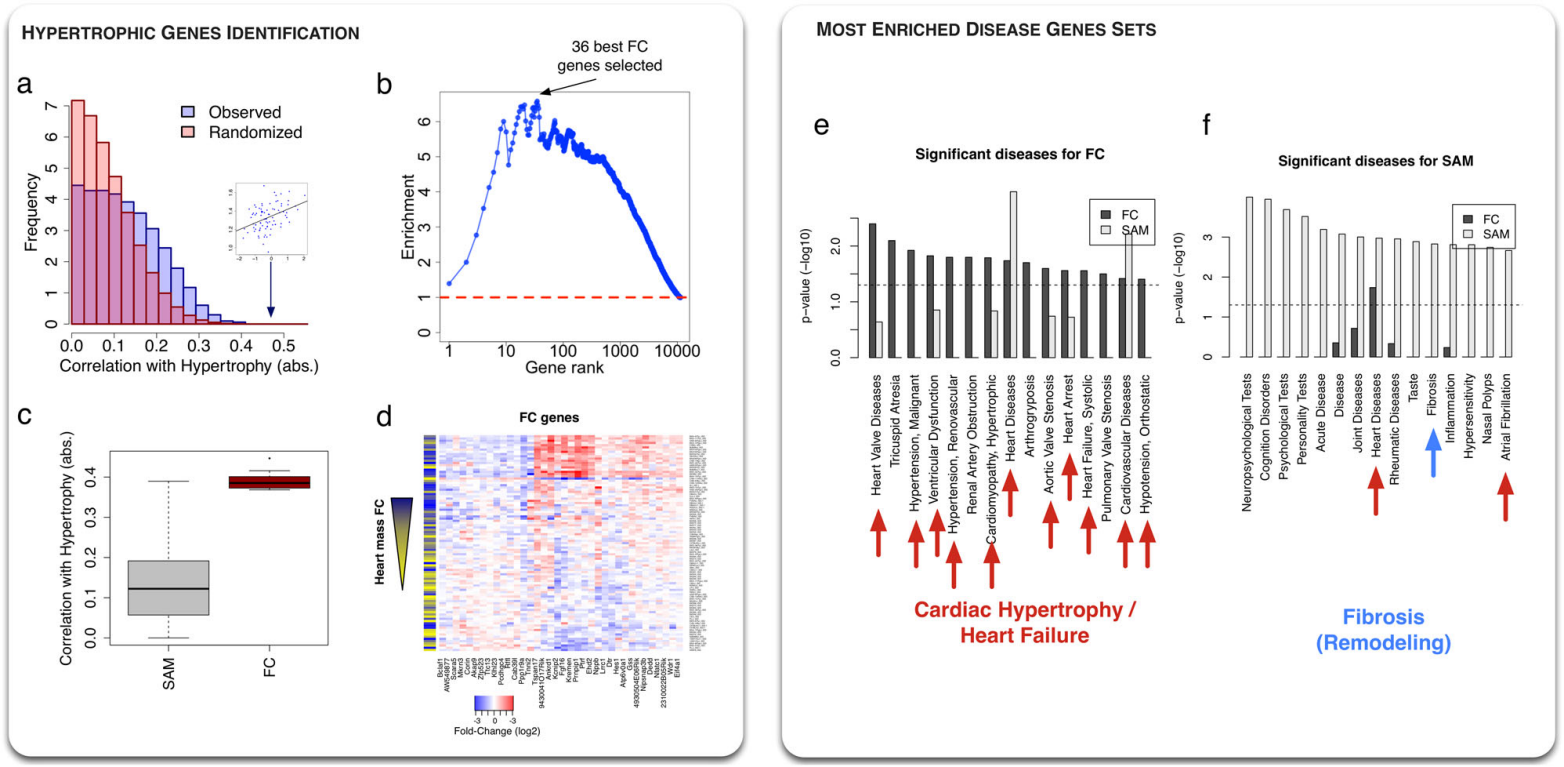
Identification of genes associated with the severity of cardiac hypertrophy. **a** Histogram of the absolute values of the correlations between the FCs of genes expression and hypertrophy for all genes (blue, observed, red, randomized phenotype). Genes individual FCs are more correlated to hypertrophy than expected. Inset plot corresponds to the best observed correlation. **b** The previous enrichment is assessed by computing the ratio of the area under the observed and randomized curve as a function of correlation cutoffs. Cutoffs are matched to the genes correlations ranked in decreasing order. The enrichment peaks at *N* = 36 genes, which defines the set of “FC” genes. **c** Boxplot comparing the values of the absolute correlation with hypertrophy for the 2538 SAM genes resulting from a population-wide DEG study (see main text) and for the 36 identified individual FC genes. FC genes have significantly higher correlation. **d** Heatmap showing the 36 genes (columns) log fold-changes across strains (rows). The left column shows the degree of hypertrophy (yellow = low, dark blue = high). Hierarchical clustering shows a natural grouping of the strains by the severity of hypertrophy. **e** Enrichment of 36 best FC genes in human disease genes from GWAS studies. The 15 most enriched sets are shown. Red arrows indicate cardiac diseases (11/15). The enrichment of the 36 best SAM genes is shown for comparison, with low enrichment in the found sets. **f** Similar than (**g**), for 36 SAM genes. These genes show enrichment in “Fibrosis”, a feature of structural remodeling during cardiac hypertrophy

Here we develop a method to determine which genes show individual, strain-specific expression FCs significantly correlated to the individual hypertrophic response measured by the individual fold-change of heart mass. We use microarray and phenotype expression data described in.^13^ Since our methodology is based on correlations, we choose to select those genes that belong to the giant component of the gene co-expression network above a certain correlation cutoff (see Methods and Figs. S1, 2). The advantages of such a filter compared to one based on absolute expression levels is that it yields a clear, well-defined cutoff (Figure S1b) while also rejecting genes having high expression but artefactual correlations (e.g., hitting the microarray saturation level in Figure S1c). We obtain a filtered set of 11,279 high-confidence genes. We then compute for all genes the absolute Pearson correlation between the gene expression fold-change and the individual hypertrophic response (Fig. 2a, blue histogram). To control for False Positives, we compute the expected correlations when randomizing the phenotype by shuffling strain labels (see Methods and Fig. 2a, red histogram). One can see significant enrichment in genes with high correlation to the trait. To quantify this enrichment, we compute the proportion of observed (blue) correlations divided by the proportion of correlations in the randomized cases (red) above various correlation cutoffs. Figure 2b shows this enrichment as a function of the gene rank, ordered by decreasing absolute value of the correlation with hypertrophy. The enrichment shows a peak at 36 genes, followed by a plateau until ~500 genes, and a subsequent decrease. We define these 36 genes as our candidates to describe the hypertrophic spectrum. These genes are listed in Table 1, along with references supporting the involvement of several of them in cardiac hypertrophy and HF. In the following, we refer to this set of genes as the “FC” set.

**Table 1:** List of genes predicted with the individual fold-change analysis

Rank	Gene	Correlation	Known role from the literature
1	Rffl	−0.446656	Hypertension^57^
2	Wdr1	0.415692	Cardiac Hypertrophy^58^
3	Nppb	0.408886	Heart failure marker^59^
4	Atp6v0a1	0.407205	Hypertension^60^
5	Ankrd1	0.406246	Dilated cardiomyopathy^61^
6	Eif4a1	0.404824	
7	Dtr (HB-EGF)	0.403043	Heart failure^62^
8	Kcnip2	0.402246	Downregulated in hypertrophy^23,24,26^
9	Pcdhgc4	−0.402053	
10	Hes1	0.400076	Heart outflow tract development.^63^ Validation in the present study.
11	4930504E06Rik	0.396189	
12	Akap9	−0.389713	LQT syndrome^64^
13	2310022B05Rik	0.3897	
14	Bclaf1	−0.388563	
15	Ttc13	−0.387981	
16	Nipsnap3b	0.387325	
17	Gss	0.386407	Glutathione synthetase, linked to cardiac abnormalities^65–67^
18	Klhl23	−0.385625	
19	Tspan17	0.384865	
20	Tnni2	−0.383516	
21	Cab39l	−0.381902	
22	Ptrf (Cavin-1)	0.381134	Dilated cardiomyopathy^68^
23	Dedd	0.378059	
24	9430041O17Rik	0.375683	
25	Fgf16	0.373829	Heart disease^69^
26	Ehd2	0.372787	Regulate cardiac membrane protein targeting.^70^ Interact with ankyrin-B (ANK2 gene whose mutation is associated with hypertrophic cardiomyopathy^71^)
27	Ppp1r9a	−0.372641	Subunit of the same complex than PPP1R3A, involved in HF in human patients^51^
28	Kremen	0.372366	Interacts with Wnt signaling^72^
			Wnt signaling also plays a role in cardiac hypertrophy^73^
29	Scara5	−0.372294	
30	Zfp523	−0.372223	
31	Nfatc1	0.371409	Cardiac hypertrophy^34^
32	Corin	−0.369546	Cardiac protease that regulates blood pressure by activating natriuretic peptides, involved in cardiac hypertrophy^74^
33	Prnpip1	0.369466	
34	Lrrc1	0.369161	
35	AW549877	−0.368865	
36	Mkrn3	−0.368269	

As a comparison, we compute the population-wide DEGs using Significance Analysis of Microarray or SAM.^19^ This exhibits 2538 DEGs at a False Discovery Rate of 1e-3 (see Methods). Interestingly, we find no significant overlap (*p* = 0.68, hypergeometric test) between these SAM genes and the FC set, with six genes common to both sets (*Tspan17*, *Ppp1r9a*, *Bclaf1*, *AW549877*, *Gss*, 2310022B05Rik, and 9430041O17Rikm). In general, correlations between the individual fold-changes of the SAM genes and the degree of hypertrophy are found to be quite low (Fig. 2c). This shows that population-wide analyses do not naturally yield genes associated to the individual strength of phenotypic change, calling for a specific method to uncover them.

The 36 FC genes are shown in Fig. 2d. As expected from the absence of overlap with SAM genes, the FC genes have both negative (blue) and positive (red) fold-change across the different strains, meaning that they have negligible average fold-change at the population level. A question that arises is whether the variability observed in the individual fold-changes of gene expression across strains is a consequence of genetic variability, or merely reflects environmental or experimental spurious effects. To investigate this question, we take advantage of the fact that gene expression has been replicated in nine strains post-ISO. Since mice from the same strain have a similar genetic background, they should therefore show very comparable individual fold-changes. Expression fold-change is shown for the 36 FC genes for the replicated strains in Figure S3a. We assess the replicability by computing the Spearman rank correlation of the 36 FC genes fold-change profiles between mice from replicated strains. We find a large mean correlation of 0.76, compared to 0.14 for pairs of strains taken at random among the non-replicated pool with a statistically very significant *p*-value (*p* = 1.6e-7, Wilcoxon test, see Figure S3b). This result shows that individual fold-changes are tightly controlled at the genetic level and that the ranking of the genes by FC is preserved for approximately 2/3 of the cases. We also assessed replicability by making a scatter plot of the log2 expression fold-change computed with the original and replicated ISO treated hearts compared to the same control heart for the 36 FC genes and 9 strains (Figure S3c). A correlation analysis of this scatter plot yields a correlation of 0.57 and very low *p*-value (*p* < 2.2e-16), confirming that individual fold-changes are predominantly genetically determined.

In the following, we wish to evaluate further the biological signal carried by these FC genes missed by population-wide methods.

### Biological relevance of the identified FC genes

Given the importance of the genetic control of those genes, they must be more susceptible to genetic variations. To explore that idea, we look at the enrichment in disease genes coming from previous GWAS. We use HuGE database of human genes associated with 2711 different diseases (see Methods). First, we convert the mouse gene names to human as described in the Methods. Then, we rank the diseases according to their enrichment in 36 FC (resp. 36 best SAM) genes using a hypergeometric test assuming as null hypothesis a uniform repartition of the genes across diseases. Results are shown in Figs. 2e, f for the 15 most enriched diseases in each case. We observe that FC genes are strongly enriched in heart diseases (11 in the 15 most enriched diseases) while SAM genes are only enriched in two cardiac diseases and in fibrosis, a feature characteristic of the structural remodeling taking place during HF.^27^ Those findings exhibit two distinct roles of FC and SAM genes in the progression of cardiac hypertrophy. While the cross-talk between cardiac fibroblasts and myocytes during cardiac hypertrophy has been studied previously,^28^ here we disentangle their relative contributions into a shared, population-wide fibroblastic component, and a fine-tuned, individualized component capable of explaining the severity of cardiac hypertrophy. Moreover, the enrichment of FC genes in human GWAS genes also highlights the relevance of the present HMDP data analysis to human cardiac hypertrophy and HF.

### Co-expression and co-regulation

**Fig. 3:**
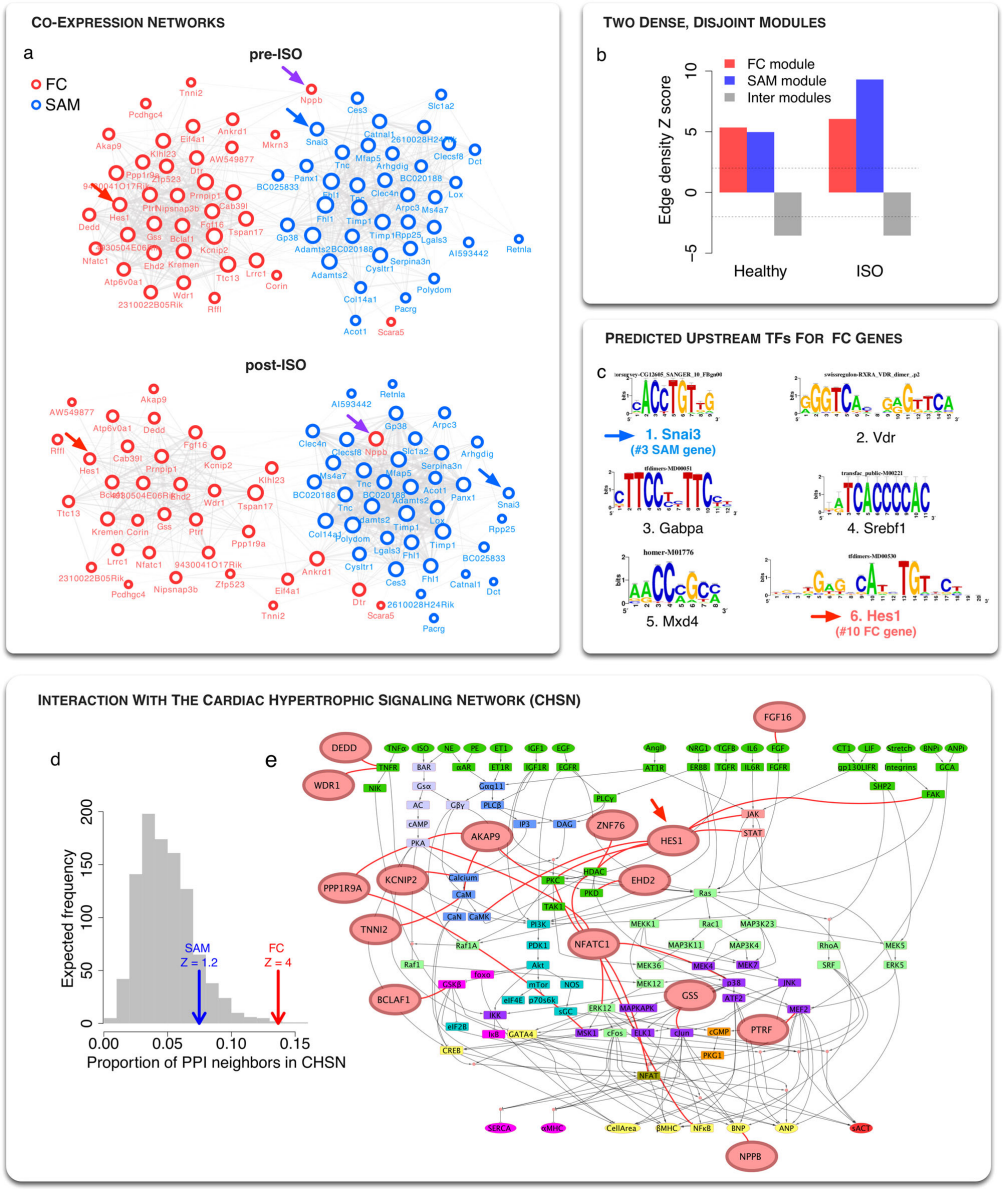
FC genes are co-regulated and significantly connected to the cardiac hypertrophy signaling network (CHSN). **a** Co-expression networks of the 36 best FC and SAM genes in healthy and post-ISO hypertrophic strains. Edges are drawn between two genes if the square Pearson correlation is greater than 0.1 (*r*^2^ > 0.1). The two modules segregate naturally using a force layout algorithm, showing that the modules have high clustering but only few links between themselves. Interestingly, Nppb (purple arrow) segregates with SAM genes, especially in ISO condition. **b** The edge density of the FC module, the SAM module, and the FC to SAM edges is computed and compared to the density expected for random sets of nodes of the same size (see Methods). The corresponding Z scores are significantly high (*Z* > 2) for both modules, indicating high co-expression. However, there are significantly fewer links than expected between the two modules (*Z* < −2), indicating that they are disjoint in the co-expression network, (**c**) List of the 6 most enriched TF motifs in the ±20 kb regions around the 36 FC genes TSSs predicted using iRegulon.^30^ Interestingly, Snai3 (blue arrow) is a SAM gene and Hes1 (red arrow) a FC gene, suggesting a crosstalk between the two modules at the gene regulatory level. **d** Proportion of neighbors in the interactome that belong to the Cardiac Hypertrophy Signaling Network or CHSN^20^ for different gene sets: the FC set (red arrow), the 36 best SAM genes (blue arrow) and 1000 realizations of random nodes in the interactome with the same size as the FC set (gray histogram). *Z*-scores are computed relative to the gray distribution. The FC set is significantly connected to the CHSN, while the SAM genes are not significantly different than a random set. **e** Network visualization of the CHSN,^56^ along with neighbors from the 36 best FC genes (red nodes). A more detailed interaction network is shown in Figure S5

The identified sets of population-level and individual FC genes have until now been considered as collections of independent genes. However, in the cell, genes function together to achieve higher-order physiological functions. Such a collective behavior can be assessed in the framework of co-expression networks, where genes are related by the similarity of their profile of expression across different conditions. In the context of the HMDP, we investigate whether the predicted sets of genes show evidence of co-regulation in healthy and post-ISO hypertrophic strains. To that extent, we compute the squared Pearson correlations (*r*^2^) between the 36 best genes of both the FC and SAM sets. Correlation matrices are then cut off at *r*^2^ > 0.1 to keep significant interactions. We show in Fig. 3a and b the resulting co-expression networks in pre and post-ISO conditions. We clearly see that the two sets of genes form dense modules, and are disconnected from each other, with only few links between the two sets. Interestingly, we see that the biomarker and modulator of hypertrophy *Nppb*^29^ acts as a bridge between the two modules in pre-ISO condition (Fig. 3a, top), and is even found strongly co-expressed with the SAM genes in post-ISO mice (Fig. 3b). This suggests a role for *Nppb* in driving a cross-talk between FC genes and SAM genes. Finally, to quantify the relative density of the modules, we compared them to 1000 sets of a similar number of randomly selected genes. We show the resulting *Z* scores in Fig. 3c. Both SAM and FC sets show much stronger co-expression than randomly expected, with the SAM module being even denser under ISO condition. On the contrary, the density of links between the two modules is significantly smaller than expected by chance, indicating that the two sets of genes are disjoint sets in the co-expression network. Overall, these results show that the FC and SAM genes form two tight, disjoint communities in the co-expression network, both in pre-ISO and post-ISO mice.

The finding that the FC genes are strongly co-expressed suggests that they are co-regulated. To explore this possibility, we look for enrichment in common TF binding sites in the vicinity of the 36 FC genes. To compute the enrichment, we use iRegulon, a recent algorithm integrating different TF motifs databases and using phylogenic conservation to identify overrepresented binding sites in the −20/ +20 kb regions around the Transcription Start Sites of genes of interest (see Methods).^30^ The identified motifs are then ranked by target enrichment among selected genes, and are associated with a list of putative TFs that can bind them (Fig. 3c). We find that the best-ranked motif is associated with repressor TFs Scrt1 and Scrt2, known to modulate the action of basic helix-loop-helix TFs.^31^ Interestingly, the corresponding PWM motif is also matched to Snai3 TF, a gene ranked 3rd among SAM genes. The 2nd motif, VDR, is known to be involved in heart failure and cardiac hypertrophy.^32^ Finally, the sixth predicted TF is associated with Hes1, which ranks 10th among the FC genes. This indicates that there is a cross-talk between the two modules at the gene regulatory level, with both FC and SAM genes being involved in the regulation of the expression of the FC genes.

### Exploration of the neighborhood in the interactome

While useful to detect gene regulatory changes involved in the disease process, gene expression does not capture post-translational changes and interactions that occur at the protein level. To explore the potential involvement of the predicted sets of genes at the protein level, we use a previously published human interactome combining high-throughput and literature curated protein–protein, metabolic, kinase–substrate, signaling and to a lesser extent regulatory interactions.^33^ After converting to human gene symbols (see Methods), the proteins encoded by the 36 best FC and SAM genes have respectively 364 and 346 interacting partners. We then compute pathway enrichment for these neighbors (see Methods). The other most highly enriched pathway is linked to NFAT signaling, known to be important in HF.^34^ Interestingly, we find that the second most enriched pathway for FC neighbors is a previously published Cardiac Hypertrophy Signaling Network (CHSN) containing 106 nodes (corresponding to 218 genes) giving a predictive model of hypertrophy in response to multiple stressors including ISO^20^ (Figure S4). Indeed, about 14% of FC neighbors are components of this network, compared to a predicted random association of 4% (*Z* = 4, Fig. 3d). The CHSN is shown in Fig. 3e and in more details in Figure S5, along with FC nodes directly interacting with CHSN nodes. In particular, we find that Hes1 is interacting with several nodes of the CHSN at different levels of the hierarchy, namely FAK, JAK, STAT, CamK, PKC, and HDAC.

### Experimental validation of Hes1

**Fig. 4:**
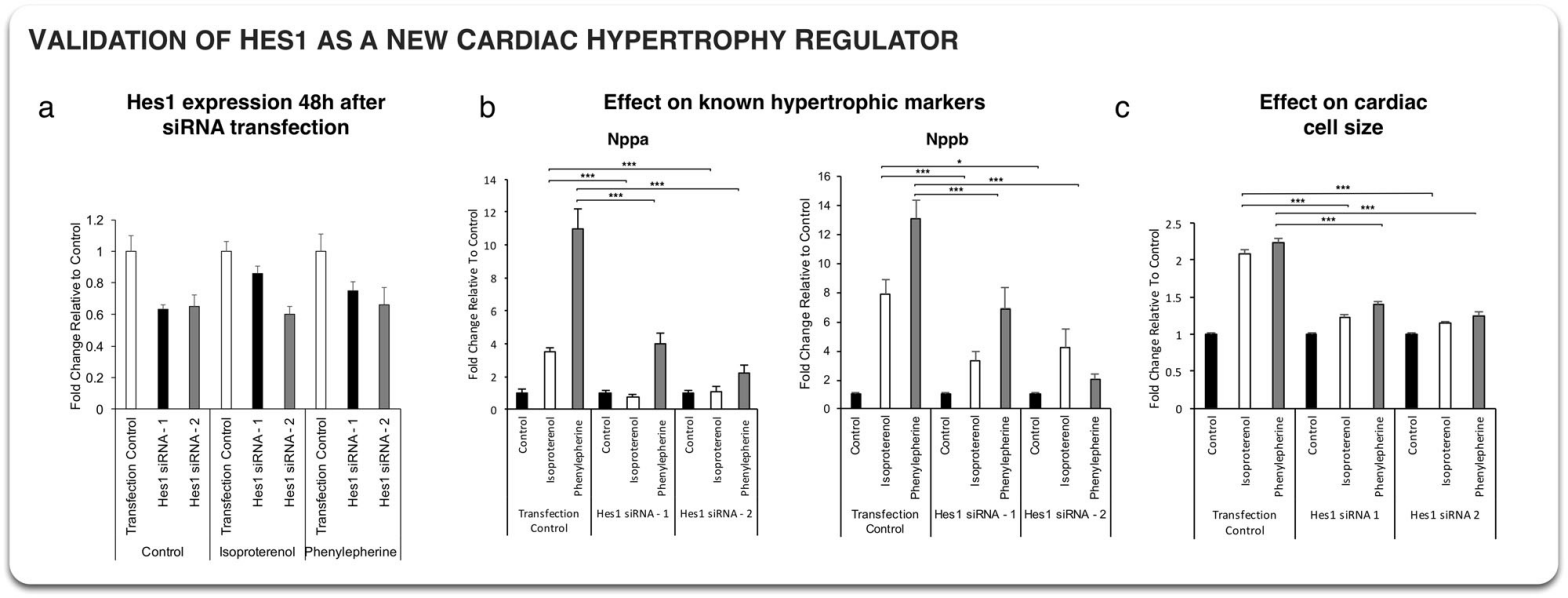
Validation of Hes1 as a cardiac hypertrophy regulator. **a** *Hes1* mRNA expression following 48 h after siRNA transfection in a control, isoproterenol or phenylephrine medium. Three siRNAs were used, a scrambled, control one and two *Hes1* specific siRNAs. Both *Hes1* siRNAs show systematic downregulation of *Hes1* mRNA in all conditions. **b** Effect of *Hes1* knockdown on the known hypertrophic makers *Nppa* and *Nppb*. In both case, *Hes1* knockdown leads to a significant change in biomarkers activation in isoproterenol and phenylephrine conditions (*p < 0.05, ***p < 1e-3, Student *t*-test). **c** Effect of Hes1 knockdown on neonatal rat ventricular myocytes size relative to control medium cell cross-sectional area. Both siRNAs lead to a drastic 80–90% decrease in hypertrophy in both isoproterenol and phenylephrine media

The previous results point toward a role for *Hes1* in cardiac hypertrophy and heart failure. Indeed, *Hes1* was found to be a FC gene, an upstream regulator of FC genes, and an interactor with several components of the CHSN. To determine the function of *Hes1* in the context of cardiac hypertrophy and heart failure, we performed siRNA knockdown in neonatal rat ventricular myocytes followed by treatment with beta-adrenergic agonist ISO or alpha-adrenergic agonist phenylephrine (PE) containing media. Both agents induce hypertrophy through different molecular pathways, as can be seen in the CHSN (see Fig. 3e). Using siRNA to silence *Hes1* expression, we achieved a 20–40% decrease in *Hes1* expression when compared to transfection control (Fig. 4a and Table S3). At the molecular level, treatment with either ISO or PE containing media drastically increases the expression of the HF markers *Nppa* and *Nppb*, which rose 3.5 and 7.9-fold, respectively under ISO treatment and 11-fold and 13-fold, under PE treatment in cells transfected with the control siRNA. Strikingly, knockdown of *Hes1* expression strongly impaired the induction of these two markers under both treatment conditions. *Nppa* induction was reduced up to 110 and 88% under ISO and PE treatment while *Nppb* induction was reduced up to 66 and 91% under ISO and PE treatment, respectively. In addition to these molecular changes, we investigated the role of Hes1 in modulating the increase in cardiomyocyte cell cross-sectional area upon treatment with ISO and/or PE. As expected, following ISO/PE treatment, cells transfected with the control siRNA doubled in cellular cross-sectional area (Fig. 4c, Figure S6, and Table S4). In comparison, cells transfected with the *Hes1* siRNA showed up to 87 and 79% reduction in cell cross-sectional area increase following treatment with ISO and PE, respectively. This effect is consistent with the fact that HMDP strains showing no or mild hypertrophy exhibit strong negative fold-change of *Hes1* (Figure S7). Taken together, these findings strongly suggest a role for *Hes1* as a regulator of cardiac hypertrophy in vitro.

## Discussion

In the present study, we investigated the spectrum of cardiac hypertrophy and HF development in 100+ genetically diverse mice from the HMDP when subjected to chronic ISO infusion. We have analyzed two types of responses. First, the global response at the population level with a large number (1000+) of genes involved, as detected by the SAM algorithm. Their global fold-change is representative of the global hypertrophy observed across all strains. However, the magnitude of their fold-change at the individual level does not predict the degree of individual hypertrophy. Using a correlation-based method, we found another group of ~40 genes that predicts the degree of hypertrophy. We named these the “FC” genes in reference of the fact that we found them using their individual, strain-specific fold-change. Surprisingly, these genes have a near zero fold-change at the population level due to the canceling contributions of up and down-regulation in different strains, so that they are not detected using classical differential expression tools. While several FC genes have previously been implicated in cardiac hypertrophy and HF (see Table 1), their high variability in such a controlled setup has not been explored previously. We showed that these genes are enriched for heart failure gene candidates previously described in the literature, as well as for human cardiac disease genes. On the other hand, the best SAM genes are enriched in fibrosis disease genes. ISO has been shown to induce first myocardial fibrosis concomitantly with myocyte necrosis, followed by myocyte hypertrophy on a longer time scale,^35^ and fibrosis is also known to be an early manifestation of hypertrophic cardiomyopathy.^36^ Our results suggest that population-level SAM genes are predominantly associated with the early fibroblast response. On the other hand, since the change of heart mass is primarily determined by myocyte growth, our results suggest that FC genes are associated with the strain-specific degree of myocyte growth induced by beta-adrenergic stimulation.

We further investigated the roles of these genes in different biological networks. We found that both FC and SAM genes form distinct co-expressed modules. Interestingly, *Nppb* (encoding the BNP protein), a widely used biomarker and modulator^29^ of HF, belongs to the FC set but is co-expressed with SAM genes in healthy mice, providing a unique bridge between the two sets. We note that this result is consistent with the previous finding that *Nppb* is an antifibrotic hormone produced by myocytes with an important role as a local regulator of ventricular remodeling in mice.^37^ Indeed, *Nppb* is correlated to the fibrotic SAM genes in healthy mice, consistent with a regulatory homeostatic behavior, but is found among FC genes after beta-adrenergic stimulation, consistent with a response proportionate to myocytes hypertrophy. It is also interesting to note that the SAM module overlaps significantly (*p* = 3.4e-6, hypergeometric test) with a co-expression module previously found in post-ISO mice and shown to be involved in cardiac hypertrophy.^38^ Indeed, it shares the genes *Timp1, Tnc, Mfap5, Col14a1* and *Adamts2*, the latter of which was validated experimentally as a regulator of cardiac hypertrophy.

We then predicted several TFs to study this co-regulation. Interestingly, among the top TFs predicted as regulators of the FC genes, one of them, *Hes1*, belongs to the FC genes, and another one, *Snai3*, belongs to the SAM genes. We note that both inhibitory (Snai3, Hes1) and activatory (Vdr, Srebf1) TFs were found to have enriched binding sites around FC genes TSSs. This suggests a potential regulatory balance that could explain the up and down-regulation observed for these genes across strains. We then looked at potential post-translational effects at the protein level by using an integrated interactome. We found that FC genes were strongly interacting with a CHSN previously shown to be predictive of cardiac hypertrophy in response to ISO and other stressors.^20^ This may indicate that several of those genes are upstream of a causal chain of events at the post-translational level that control myocyte growth. We note that the FC gene *Nppb* is present both as an input and an output of the CHSN. This exemplifies an interesting feedback architecture where downstream effects can causally affect upstream regulation. Overall, the FC genes constitute a HF “disease module” formed of co-regulated genes connected to the CHSN at the protein level.

A key finding of our study is that there is strong strain-to-strain variation in response to a stressor under similar well-controlled environmental conditions. This variation is largely explained by the different genetic backgrounds, as shown by the consistent responses in mice from same strains (Figure S3) and the strong enrichment in heart diseases GWAS (Fig. 2e). For example, *Kcnip2* is known to be downregulated concomitantly with a reduction of *I*_to_ magnitude in cardiac hypertrophy.^24,26^ Our results are consistent with this finding for the previously studied 129 × 1/SvJ strain,^24^ but show that *Kcnip2* is upregulated in many strains with pronounced hypertrophy leading to an overall positive correlation between *Kcnip2* expression and heart mass FC. This indicates that there are multiple possible compensatory mechanisms underlying a similar patho-phenotype. Similarly, we observed strong variation in the fold-change of *Nppb*. It was previously shown to be over-expressed during cardiac hypertrophy as an anti-fibrotic factor.^29^ Using our multiple strains setup, we observed a positive correlation between *Nppb* change of expression and the degree of hypertrophy. However, we also observed some cases were hypertrophic strains exhibit down-regulation of *Nppb*, including the widely used C57BL/6 J and 129 × 1/SvJ strains (see Fig. 2d and S3).

Finally, our approach was validated by testing *Hes1*’s role in cardiac hypertrophy. *Hes1* was chosen because of its involvement at different levels: found as a FC gene, *Hes1* is also a predicted TF regulating the FC genes and a key interactor of the CHSN. *Hes1* is part of the Notch signaling pathway which is highly conserved and involved in cell-cell communication between adjacent cells.^39^ This pathway is well known to play a crucial role in cardiac development and disease. Notch activity is required in complex organs like the heart that necessitate the coordinated development of multiple parts.^40^ Specifically, functional studies have shown that Notch activity is required for cardiovascular development and that Notch signaling causes downstream effects such as cell fate specification, cell proliferation, progenitor cell maintenance, apoptosis, and boundary formation.^39^ In previous studies, *Hes1* expression was observed to increase following myocardial infarction and other ischemic cardiomyopathies. Increased expression of *Hes1* was also shown to inhibit apoptosis of cardiomyocytes and promote instead their viability. However, whether *Hes1* acts as a regulator of heart failure markers has remained unclear.^41^ Here, we show that *Hes1* knock-down induces a dramatic reduction of hypertrophy by 80–90% (Fig. 4c), identifying for the first time *Hes1* as a key regulator of cardiac hypertrophy. Importantly, this result is consistent with the HMDP, where strains with no or mild hypertrophy have 20–50% decrease in *Hes1* after ISO injection (Figure S7b).

Overall, we have explored the individual, strain-specific responses to stressor-induced HF and identify 36 FC genes that are missed by traditional population-wide methods of DEG analysis. We have shown that these FC genes provide a completely distinct, albeit complementary, picture of HF than population-wide DEGs. In particular, FC genes are enriched in human cardiac disease genes and hypertrophic pathways. This is important since previous studies that use population-level methods to identify DEGs have concluded that murine models are of limited relevance to human HF.^42,43^ In contrast, our findings show that FC genes, identified by a personalized differential expression analysis in a genetically diverse population of mice, are relevant to human HF. By linking those genes both to upstream regulators and to a signaling network predictive of cardiac hypertrophy, we provide new insights into the regulation of the severity of and resistance to cardiac hypertrophy at the individual level, and validate *Hes1* as a regulator of cardiac hypertrophy in vitro. We believe this approach to be critically important for the appropriate design of upcoming experiments directed at unraveling causal genes in complex diseases.

## Methods

### Overview of the HMDP

The HMDP consists of a population of over 100 inbred mouse strains selected for usage in systematic genetic analyses of complex traits. Strain were selected to increase resolution of genetic mapping with a renewable resource that is available to all investigators worldwide as well as to create a shared data repository that would allow the integration of data across multiple scales, including genomic, transcriptomic, metabolomic, proteomic, and clinical phenotypes. The core of our panel for association mapping^44–46^ consists of 29 classic parental inbred strains which are a subset of a group of mice commonly called the mouse diversity panel. HMDP strains were chosen by eliminating closely related strains and removing wild-derived strains. The decision to remove wild-derived stains reflects a tradeoff between statistical power and genetic diversity. While leaving out wild-derived strains sacrifices genetic diversity to some degree, the HMDP increased the statistical power (assuming the same number of animals) to identify genetic variants polymorphic among the classical inbred strains which affect traits. These variants yield a tremendous amount of phenotypic diversity among the classical inbred strains.

### ISO treatment

As previously described,^13,47^ 30 mg per kg body weight per day of ISO was administered for 21 days in 8–10 week old female mice using ALZET osmotic mini-pumps, which were surgically implanted intraperitoneally. All animal experiments were conducted following guidelines established and approved by the University of California, Los Angeles Institutional Animal Care and Use Committee (IACUC) and housed in an IACUC-approved vivarium with daily monitoring by vivarium personnel.

### Hypertrophy measurement

As each mouse in a strain is genetically identical, we used several mice from the same strain for measuring the cardiac hypertrophic response to ISO treatment. More specifically, we used on average three untreated mice serving as control hearts and about three ISO treated mice of the same strain to measure the cardiac hypertrophic response. This response was studied in a total of 104 genetically different strains with the precise number of control and treated hearts for each strain given in Table S1. The number of untreated control hearts per strain was 2.75. The average number of ISO treated hearts per strain was 3.5. At sacrifice, hearts were excised, drained of excess blood and weighed. Each of the four chambers of the heart (left ventricle with inter-ventricular septum, right-ventricular free wall, right and left atria) was isolated and subsequently weighed. Cardiac hypertrophy for a given strain was calculated as the increase in average total heart weight after ISO treatment compared to control mice.

### Heart biopsy for microarray analysis

As for the hypertrophy measurement, we exploited the fact that each mouse in a strain is genetically identical to extract heart tissue for microarray analysis in both untreated and ISO treated mice from the same strain. The left ventricle of each heart was cut into quarters with each piece weighing on average about ±25 mg a few mg depending on the amount of hypertrophy and two pieces were used for microarray data analysis. Due to the large number of strains analyzed and the cost of microarray data analysis, we used one untreated control heart and one ISO treated heart per strain for about 90% of the strains. However, since mice of a given strain are renewable, the HMPD offers the possibility to use triplets, quadruplets, and higher multiples of isogenic subjects for experimentation. This feature was used to measure gene expression in replicates (e.g., two hearts in control or two hearts after ISO treatment) to test for replicability in ~10% of the strains (9 strains analyzed in Figure S3).

### Microarray data analysis

Following homogenization of left ventricular tissue samples in QIAzol, RNA was extracted using the Qiagen miRNAeasy extraction kit, and verified as having a RIN > 7 by Agilent Bioanalyzer. Two RNA samples were pooled for each strain and experimental condition and arrayed on Illumina Mouse Reference 8 version 2.0 chips. Analysis was conducted using the Neqc algorithm included in the limma R package^48^ and batch effects addressed using COMbat.^49^ In designing our study, we were cautious and distributed the treated and control conditions evenly across our three batches as well as endeavoring to include a diverse set of genetic backgrounds in each batch. Thus, we do not believe that our data suffer from the potential batch artifacts as reported in.^50^

### Overview of the gene correlation method

Traditional analyses of differential gene expression for complex diseases rely on gene expression data for two populations: a control population and a diseased (or drug treated) population. For example, in the case of HF, the control population consists of N donors with healthy hearts intended to be used for transplantation, which are biopsied for gene expression analysis when left unused, and the diseased population consists of M late stage heart failure patients whose hearts are explanted and then biopsied for gene expression analysis. Importantly, the subjects in the control and diseased population are all genetically different. Hence, if we label the subjects by $S_i$, where the index $i$ refers to subject $i$ with its own genetic background distinct from all other subjects, the $N$ subjects in the control population are $(S_1,S_2,....,S_N)$ (control subjects) and the $M$ subjects in the diseased population are $(S_{N + 1},S_{N + 2},....,S_{N + M})$ (diseased subjects).

The data sets used for the differential gene expression analysis consists then of the expression level (log2 mRNA number) of a large number of *K* genes for each subject. *K* is typically in the range of several thousands, and thus much larger than the number of control or diseased subjects (*N* or *M*, respectively) that are at most a few hundreds in the most extensive studies to date,^51^ and only a few subjects in each population in earlier studies.^7^ Let us label the expression levels by $E_i(j)$ where the subscript *i* refers to subject *i* and the index $j = 1,K$ refers to gene *j*. To find out if a given gene *j* among the *K* genes is differentially expressed, it suffices to use a standard statistical test analogous to a student *t*-test to decide if the gene expression data for the control group $\left( {\log _2E_1\left( j \right),\log _2E_2\left( j \right),\, \ldots \,,\log _2E_N\left( j \right)} \right)$ (expression data for gene *j* in control population) and for the diseased group $(\log _2E_{N + 1}\left( j \right),\log _2E_{N + 2}(j),\, \ldots \,,\log _2E_{N + M}(j))$ (expression data for gene *j* in genetically distinct diseased population) have statistically distinguishable mean values. We note that we use the log2 of gene expression here. Indeed, raw gene expression levels measured from microarray fluorescence intensity typically have a skewed log-normal distribution resulting from a multiplicative error during the amplification process. The log transformation allows to normalize the data distribution and use classical parametric statistics such as the *t*-test for analysis. This test is carried out for all *K* genes and differentially expressed genes are then ranked in order of statistical significance (e.g., with increasing *p*-value less than some threshold of statistical significance). This approach is well-established and can be performed using existing bioinformatics tools such as SAM (Statistical Analysis of Microarrays).^19^

Because mice from the same strains are isogenic and renewable, the HMDP offers the possibility to analyze differential gene expression in a different and unique setting where subjects in the control and diseased populations have the same genetic background. The control population consists of one mouse per strain (for *N* strains) before treatment with a beta-adrenergic agonist isoproterenol (ISO) inducing cardiac hypertrophy and heart failure. Since all strains are genetically distinct the subjects in the control population are genetically distinct and can be labeled as $(S_1,S_2,...,S_N)$ (genetically identical control and diseased populations in the HMDP). Hearts from those subjects before ISO treatment are biopsied and used for microarray analysis. Biopsy requires sacrificing the animals that cannot be ISO treated. However, another mouse from the same strain can be ISO treated and similarly for all *N* strains. Therefore, the diseased/treated population is genetically identical to the control population and has the same degree of genetic diversity.

From the gene expression data alone, we can then perform the standard SAM type of differential gene expression analysis that consists of deciding if the gene expression data before $\left( {\log _2E_1\left( j \right),\log _2E_2\left( j \right),\, \ldots \,,\log _2E_N\left( j \right)} \right)$ (expression data for gene $j$ in control strains) and after $\left( {\log _2E_1^\prime \left( j \right),\log _2E_2^\prime \left( j \right),\, \ldots \,,\,\log _2E_N^\prime \left( j \right)} \right)$ (expression data for gene $j$ in genetically identical treated strains) treatment have statistically distinguishable mean values, where $E_i\left( j \right)$ and $E_i^\prime \left( j \right)$ are the expression levels of gene $j$ for the isogenic subjects $S_i$ before (in control) and after ISO treatment, respectively. To do so, SAM uses a statistics based on the ratio of change in gene expression to standard deviation in the data for that gene, yielding the “relative difference”:^19^

3 $${\boldsymbol{d}}\left( {\boldsymbol{j}} \right) = \frac{{{\boldsymbol{\mu }}_{\boldsymbol{j}}^\prime - {\boldsymbol{\mu }}_{\boldsymbol{j}}}}{{{\boldsymbol{s}}\left( {\boldsymbol{j}} \right) + {\boldsymbol{s}}_0}}$$

where $\mu _j$ and $\mu _j^\prime$ are defined as the average levels of expression for gene *j* in control and ISO treatment, respectively, and the denominator $s\left( j \right) + s_0$ is the gene expression scatter as defined in.^19^ Genes that show a difference of average expression levels across both conditions that is significantly larger than their condition-specific scatters are selected and referred to as SAM genes.

One can also perform an entirely different type of differential gene expression analysis owing to the fact that, in addition to control and treated subjects belonging to the same strain having the same genetic background, the change of heart mass in response to ISO, i.e., the ratio $m_i^\prime /m_i$ of total heart mass before ($m_i$) and after ISO treatment ($m_i^\prime$) for strain i, can be measured for all strains $(i = 1,2,...,N)$ to assess the degree of hypertrophy among different strains. This ratio is calculated by measuring total heart mass for several mice from the same strain before and after ISO treatment and averaging measured values before and after ISO treatment prior to taking their ratio. Importantly, values of $m_i^\prime /m_i$ range continuously from about 1 (no change of heart mass) to 2 (two-fold change of heart mass) among strains. Differential gene expression can then be examined by asking whether a given gene $j$ contributes to the severity of cardiac hypertrophy. This can be readily done by calculating the coefficient of correlation .. (e.g., Pearson or Spearman) between the strain-specific fold change of expression of gene $j$ in response to ISO treatment among different strains $F_j = \left( {\log _2\frac{{E_1^\prime \left( j \right)}}{{E_1\left( j \right)}},\log _2\frac{{E_2^\prime \left( j \right)}}{{E_2\left( j \right)}}, \ldots ,\log _2\frac{{E_N^\prime \left( j \right)}}{{E_N\left( j \right)}}} \right)$ and the strain-specific change of heart mass among different strains $F_m = \left( {\log _2\frac{{m_1^\prime }}{{m_1}},{\mathrm{log}}_2\frac{{m_2^\prime }}{{m_2}}, \ldots ,{\mathrm{log}}_2\frac{{m_N^\prime }}{{m_N}}} \right)$. We note that for consistency with the gene expression we also used the log-ratio of phenotypic change. In our case, we use the Pearson correlation and compute:

4 $${\boldsymbol{C}}_{\boldsymbol{j}} = \frac{\langle {{\boldsymbol{F}}_{\boldsymbol{j}}{\boldsymbol{F}}_{\boldsymbol{m}}\rangle - \langle{\boldsymbol{F}}_{\boldsymbol{j}}\rangle \langle{\boldsymbol{F}}_{\boldsymbol{m}}}\rangle}{{{\boldsymbol{\sigma }}_{{\boldsymbol{F}}_{\boldsymbol{j}}}{\boldsymbol{\sigma }}_{{\boldsymbol{F}}_{\boldsymbol{m}}}}}$$

where *σ* denotes the standard deviation and 〈 〉 the average. Using that language, we note that the relative difference used for SAM genes can be rewritten as:

5 $${\boldsymbol{d}}\left( {\boldsymbol{j}} \right) = \frac{\langle{{\boldsymbol{F}}_{\boldsymbol{j}}}\rangle}{{{\boldsymbol{s}}\left( {\boldsymbol{j}} \right) + {\boldsymbol{s}}_0}}$$

This readily shows that SAM genes do not consider the strength of phenotypic change, but rely on the average gene expression change $\langle {F_j} \rangle$, while FC genes reflect how gene expression change affects phenotype change through the interaction term $\langle {F_jF_m} \rangle.$ Clearly, this correlation coefficient cannot be calculated in the setting of traditional clinical studies since the fold change of gene expression or heart mass of subjects with different genetic background is meaningless. Calculating this correlation would require to use a population of identical twins in which one twin for each pair of twins is a heart donor and the other twin is a late stage HF patient, and donor and explanted hearts could be biopsied.

The HMDP provides the experimental tool to carry out this identical twins experiment to measure expression data and trait (heart mass) for the same genetic background under different conditions (before and after ISO treatment). The correlation coefficients $C_j$ can be positive or negative and the magnitude of $C_j$ can be used to identify genes and classify them in order of statistical significance assessed by comparing $C_j$ values computed with actual data to those computed with a randomized data set (e.g., a set obtained by permuting the strain labels). We refer to genes identified by this method as FC genes to reflect the fact that they are obtained by correlating the individual fold-change of gene expression for all strains ($F_j$) with the individual fold-change of heart mass for all strains ($F_m$).

In this conceptual “identical twin” experiment, only two mice per strain are used for microarray data analysis in 90% of the strains (one control mouse and one treated mouse). This experimental limitation stems from the large number of hearts (over 200) that need to be biopsied and analyzed for gene expression. However, since mice of a given strain are renewable, the HMPD offers the possibility to use triplets, quadruplets, and higher multiples of isogenic subjects for experimentation. This feature was used to measure gene expression in replicates (e.g., two hearts in control or two hearts after ISO treatment) to test for replicability in ~10% of the strains. The results of this replicability analysis shows that genetics play a dominant role in controlling gene expression and that using two mice per strain (on in the control group and one in the treated group) is sufficient to identify FC genes. This conclusion is further supported by the fact that, remarkably, the FC genes turn out to be for the most part completely different than the traditional SAM genes, and causally related to hypertrophy as assessed by further analysis of pathway enrichment and direct experimental validation of the role of one FC gene.

### Pre-filtering of the data

In order to reduce false positive predictions and computational time, we first filtered the 25,697 genes expression data. Instead of setting an arbitrary cutoff based on the level of expression as is commonly done, we decided to use a network approach that is consistent with the correlation-based methods used in this study. The idea is that the different genotypic backgrounds across strains lead to global gene expression modulation, thus creating correlation between expressed genes. Genes not associated with the core of varying genes should be the ones that carry too much experimental noise due to low expression or systematic biases.

We first computed the absolute Pearson correlation of gene expression fold-change between all pairs of genes. This creates a complete weighted network containing all genes. We then reasoned that genes for which expression is noisy because of low expression or experimental artifacts should have a low association to the other genes. We therefore looked at the size of the Largest Connected Component (LCC) of the network when hard-thresholding with several correlation cutoffs (figure S1a). We observed a fast decrease of the LCC size at low thresholds of 0.35–0.45, followed by milder steady decrease. The derivative of this curve is presented in figure S1b, showing a strong initial trough corresponding to noisy “satellite” nodes being cut from the LCC, followed by stabilization. We chose a cutoff of 0.5 corresponding to that stabilization plateau and kept the 11,279 genes in the LCC. The effect of this filter is made clear by looking at a selection of functional genes linked to the electromechanical coupling in heart cells (figure S1c). The rejected genes (gray bars) have either low expression (e.g., *Calm4*, *Kcnd3*) or display systematic saturation effects inherent to the microarray assay, which results in noisy correlations (e.g., *Tnnc1*, *Atp2a2*). More generally, we show in Fig S2 that filtered out genes show a correlation profile with hypertrophy similar to the one expected at random. In this paper, we use these 11,279 genes as input to the different methods.

### Computation of randomized correlations

To compute the expected correlations of Fig. 2a, we first shuffle the heart mass fold-changes among strains. We then compute the correlations between all genes FCs and this randomized phenotype. We repeat that step 1000 times. The final histogram is the average over the 1000 randomizations.

### Computation of population-wide DEGs

The population-wide DEGs are computed by using Significance Analysis of Microarray or SAM^19^ between the post-ISO and the pre-ISO expression data. Using a False Discovery Rate of 1e-3, we find 2538 significant DEGs.

### Conversion from mouse symbols to human entrez IDs

In order to compute pathway and disease genes enrichment, we first needed to compute a table converting mouse gene symbols to human entrez IDs. We used UCSC genome browser mm9.kgXref, mm9.hgBlastTab and hg19.kgXref conversion tables available on the mySQL host genome-mysql.cse.ucsc.edu. The kgXref tables were used for conversion between symbols and entrez IDs while the Blast table was used to get the human orthologs of mouse genes.

### HuGE database

Disease genes were taken from the HuGE database of published GWAS genes,^52^ with a total of 2711 diseases. HF related diseases were filtered out using keywords “heart,” “cardi,” “hypert,” “aort,” “fibro.”

### Pathways

Pathways were taken from MSigDB v3.1^53^ and Wikipathways,^54^ with a total of 8690 sets of genes. A group of 106 genes corresponding to a previously published CHSN^20^ was added under the name “SAUCERMAN_cardiac_hypertrophy_pathway.”

### TF enrichment

The cytoscape plugin iRegulon^30^ was used to predict putative upstream TF regulating the studied sets of genes. Default parameters were used: 9713 PWMs scanning 20 kb centered around TSS.

### Computation of statistics

All statistics (correlations, *t*-test, Wilcoxon test, hypergeometric test) were computed using R. Hierarchical clustering was performed using default parameters of the R hclust function. *Z* scores correspond to the number of standard deviations a given observation is away from the mean of the null (random) distribution and are computed as follow:

6 $${\boldsymbol{Z}} = \frac{{{\boldsymbol{x}} - < {\boldsymbol{X}} > }}{{ < \left( {{\boldsymbol{X}} - < {\boldsymbol{X}} > } \right)^2 > }}$$

where *x* is the observed value, *X* is a set of random predictions, and < . > denotes the average.

### Cell Culture and Treatments

Right ventricular myocytes were isolated and cultured, as reported^55^ using 2–4 day old rats. Myocytes and fibroblasts were separated with Percoll density gradient. For knockdown experiments cells were transfected with *Hes1* siRNA using lipofectamine RNAimax (life technologies).

### RNA Isolation and qPCR

RNA isolation from cells was performed using Qiazol lysis reagent. cDNA synthesis was performed using the High Capacity Reverse Transcription cDNA Kit (Life Technologies). qPCR was performed using the LightCycler 480 (Roche). The number of replicates per condition is shown in Supplementary Table S2, with values ranging from 6 to 9.

### Quantification of cardiomyocyte cell cross-sectional area

Quantification of cardiomyocyte cell cross-sectional area was done following transfection with either control or Hes1 siRNA and a 48 h treatment with control or isoproterenol or phenylepherine containing media. Images were taken on a Nikon Eclipse TE2000-U microscope. Images were analyzed using the Nikon Imagine System (NIS). A total of 150 cells were used to compute the SEM.

### Code availability

Source codes are available for the community: https://github.com/msantolini/FC.

### Data availability

Microarray data may be accessed at the Gene Expression Omnibus using accession ID: GSE48760. All phenotypic and expression data may also be accessed at https://systems.genetics.ucla.edu/data/hmdp_hypertrophy_heart_failure

## Electronic supplementary material

Supplementary material

Table S1

Table S2

Table S3

Table S4

## Electronic supplementary material

**Supplementary information** accompanies the paper on the *npj Systems Biology and Applications* website (10.1038/s41540-018-0046-3).
