# Supplementary material for: A personalized, multiomics approach identifies genes involved in cardiac hypertrophy and heart failure
Source: NPJ Syst Biol Appl. 2018 Feb 24;4:12. doi: 10.1038/s41540-018-0046-3 (PMC5825397; doi:10.1038/s41540-018-0046-3)

Figure S1. A network approach to expression filtering

a. A Pearson correlation co-expression network of the genes FCs is constructed. Links with absolute value smaller than a given cutoff are eliminated, and the Largest Connected Component (LCC) size is computed. A strong LCC size decrease is observed around a cutoff of 0.4.

b. The derivative of the previous plot is shown. The cutoff of 0.5 corresponding to the end of the drop-off is selected to define 11,279 genes in the LCC.

c. Examples of cardiac functionally relevant genes in and out of the LCC. Genes left out have either low expression (*Calm4*) or saturation issues (*Tnnc1*) which introduce noise in the correlation process.

d. Effect of thresholding on network clustering. We computed the number of nodes in every connected component in the co-expression networks and ranked them by decreasing size. We show the connected component size as a function of their rank. The LCC is chosen as the filtered set of genes. The second largest connected component is of size 4. We see that there is no "competing" connected component that could contain other genes of interest.

Figure S2. Effect of the pre-filtering on FC genes

Same plots than Figure 2a, using the filtered set of genes (a), or the genes filtered out by the method from Figure S1 (b). This shows that the genes highly correlated to hypertrophy are extracted during the filtering process, while genes with low correlations are filtered out.

Figure S3 Replicability of individual fold-changes

a. Heatmap showing the fold-changes of the 36 hypertrophic genes for pairs of mice from 9 replicated strains.

b. The quality of replication is assessed by computing the Spearman correlation between the fold-changes of the 36 genes between replicated strains (red) and for random pairs of non-replicated strains (gray). The average correlation is 0.76, which is significantly higher than between two distinct strains where it is 0.14 (p=1.6e-7, Wilcoxon test).

c. Scatterplot corresponding to the data from the heatmap in a. Points correspond to personal FCs in a given strain between two replicates. We found a Spearman correlation of rho=0.53, highly significant (p<1e-16).

Figure S4 Pathway enrichment of neighbor genes in the interactome

a,b. Pathway enrichment was assessed on first neighbor genes of the FC (a) and SAM (b) genes in the human Protein-Protein Interactome [33]. The 10 most enriched pathways are shown. FC neighbors are seen to be highly enriched in a previously published Cardiac Hypertrophy Signaling Network or CHSN [20].

Figure S5. FC genes interaction with the CHSN

Network visualization of the CHSN (gray nodes) and their first neighbors in the FC set (red) in the interactome [33]. Darker nodes from CHSN indicate interaction with a FC protein.

Figure S6. Microscopic images of *Hes1* siRNA assays in neonatal rat ventricular myocytes size

Microscopic images of neonatal rat cardiomyocytes following transfection with either control or Hes1 siRNAs and a 48 hour treatment with control or isoproterenol or phenylepherine containing media. Scale bars show 100 μm.

Figure S7. *Hes1* in the HMDP

Barplots comparing Heart mass (a) and *Hes1* (b) fold-changes across HMDP strains as in Figure 1h,k. Strains with no or mild hypertrophy show a negative fold-change of *Hes1*, an effect consistent with the *Hes1* siRNA assay in neonatal rat cells of Figure 4c.

Table S1. List of 104 strains and numbers of untreated control animals and ISO treated animals used for each strain for cardiac hypertrophy measurements.

Table S2. Number of qPCR replicate experiments following *Hes1* or control siRNA transfection in neonatal rat ventricular myocytes.

Table S3. qPCR data for Hes1 and Hes1 targets corresponding to Figure 4a,b.

Table S4. Cell cross-sectional area corresponding to Figure 4c.

Figure S1

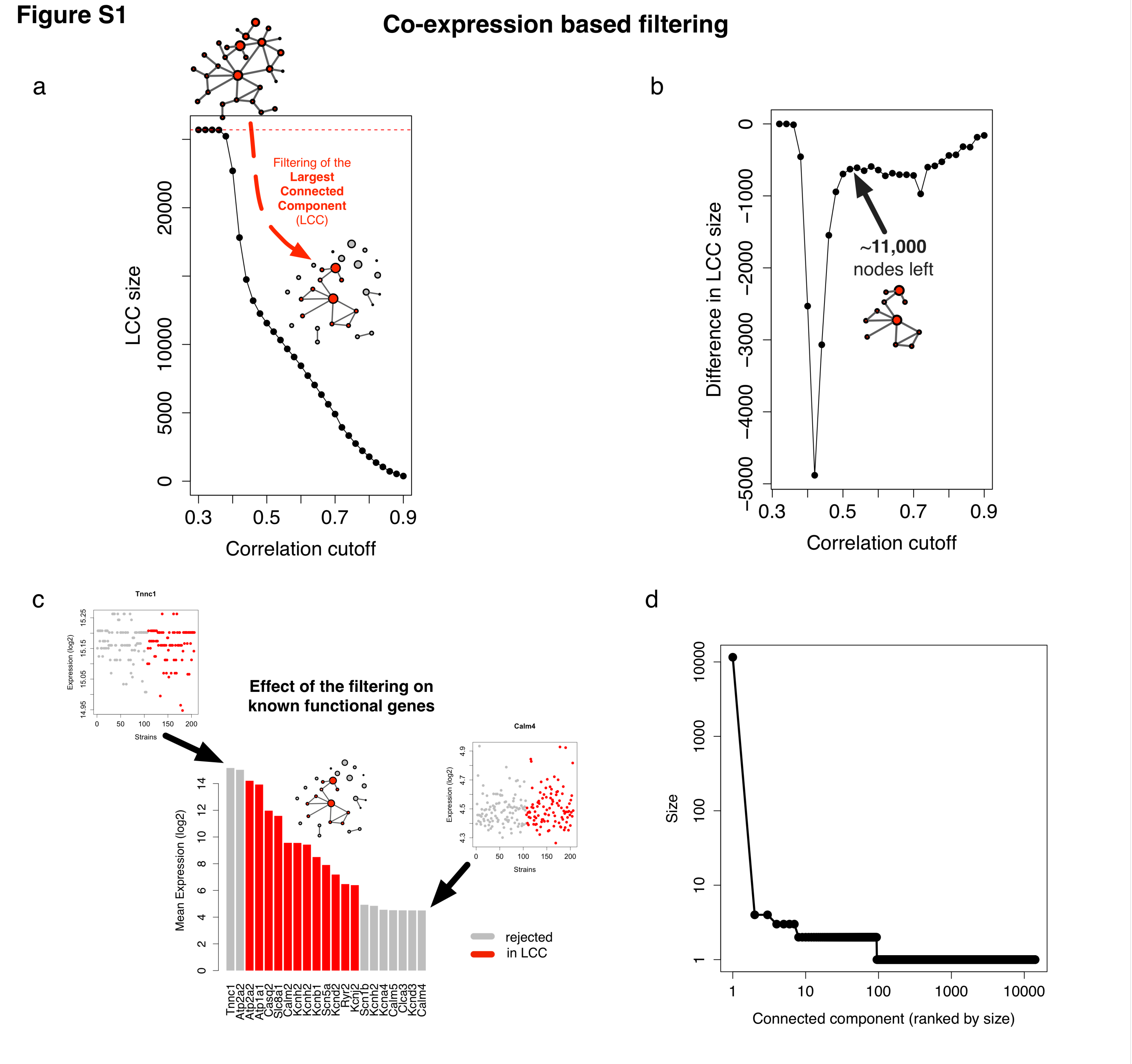

Co-expression based filtering
a
Filtering of the
Largest
Connected
Component
(LCC)
LCC size
Correlation cutoff
b
~11,000
nodes left
Difference in LCC size
Correlation cutoff
c
Effect of the filtering on
known functional genes
Tnnc1
Expression (log2)
Strains
Calm4
Mean Expression (log2)
rejected
in LCC
Tnnc1
Atp2a2
Atp2a2
Atp1a1
Casq2
Slc8a1
Calm2
Kcnh2
Kcnh2
Kcnb1
Scn5a
Kcnd2
Ryr2
Kcnj2
Scn1b
Kcnh2
Kcna4
Calm5
Clca3
Kcnd3
Calm4
d
Size
Connected component (ranked by size)

Figure S2

Effect of the pre-filtering on FC genes

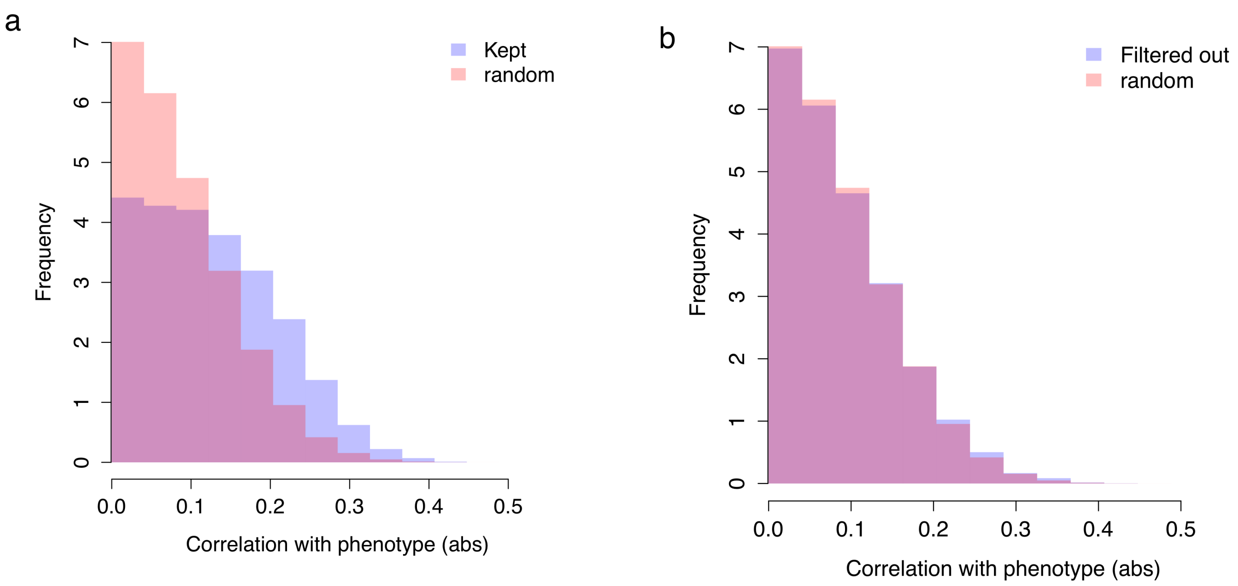

Figure S3

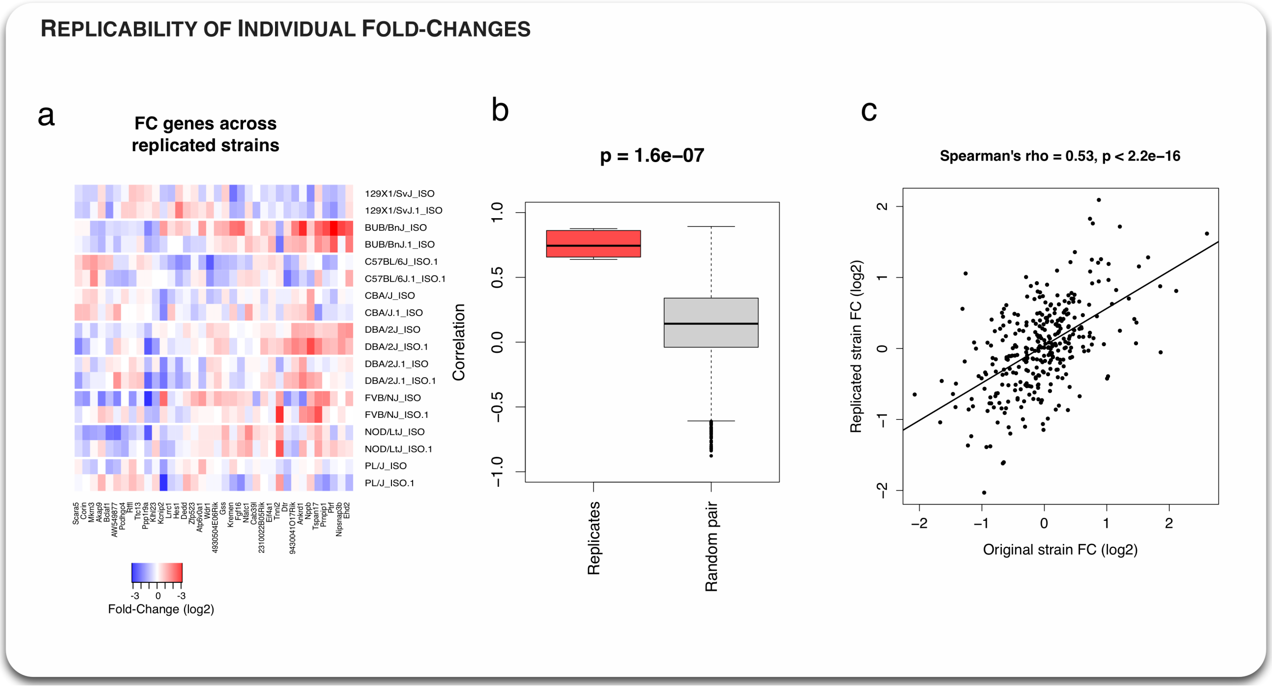
REPLICABILITY OF INDIVIDUAL FOLD-CHANGES
a
FC genes across replicated strains
129X1/SvJ_ISO
129X1/SvJ.1_ISO
BUB/BnJ_ISO
BUB/BnJ.1_ISO
C57BL/6J_ISO.1
C57BL/6J.1_ISO.1
CBA/J_ISO
CBA/J.1_ISO
DBA/2J_ISO
DBA/2J_ISO.1
DBA/2J.1_ISO
DBA/2J.1_ISO.1
FVB/NJ_ISO
FVB/NJ_ISO.1
NOD/LtJ_ISO
NOD/LtJ_ISO.1
PL/J_ISO
PL/J_ISO.1
-3 0 3
Fold-Change (log2)
b
p = 1.6e−07
Correlation
1.0
0.5
0.0
−0.5
−1.0
Replicates
Random pair
c
Spearman's rho = 0.53, p < 2.2e−16
Replicated strain FC (log2)
Original strain FC (log2)
−2
−1
0
1
2

Figure S4

Pathway enrichment of PPI neighbors

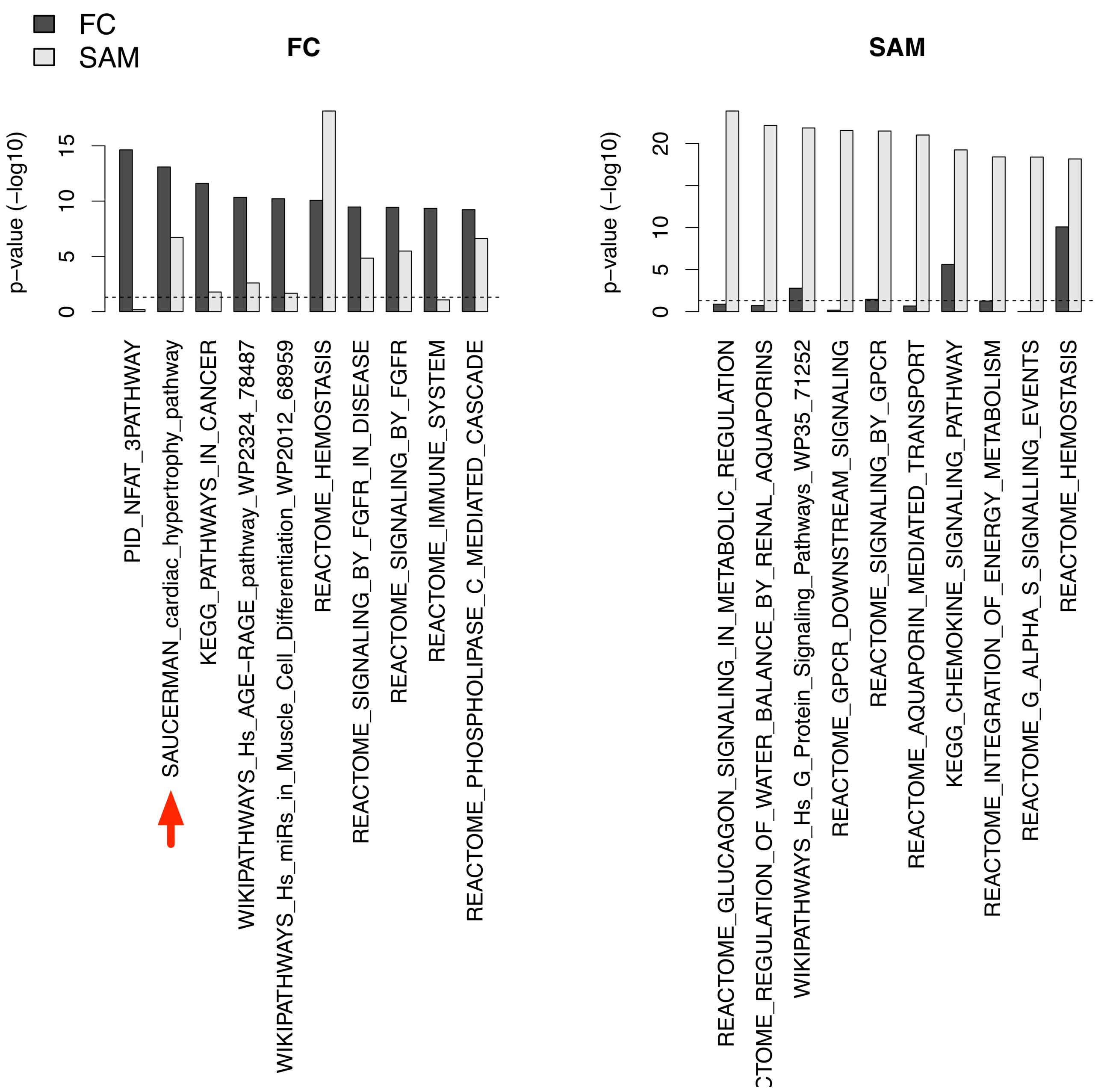

Figure S5

FC genes interaction with the CHSN

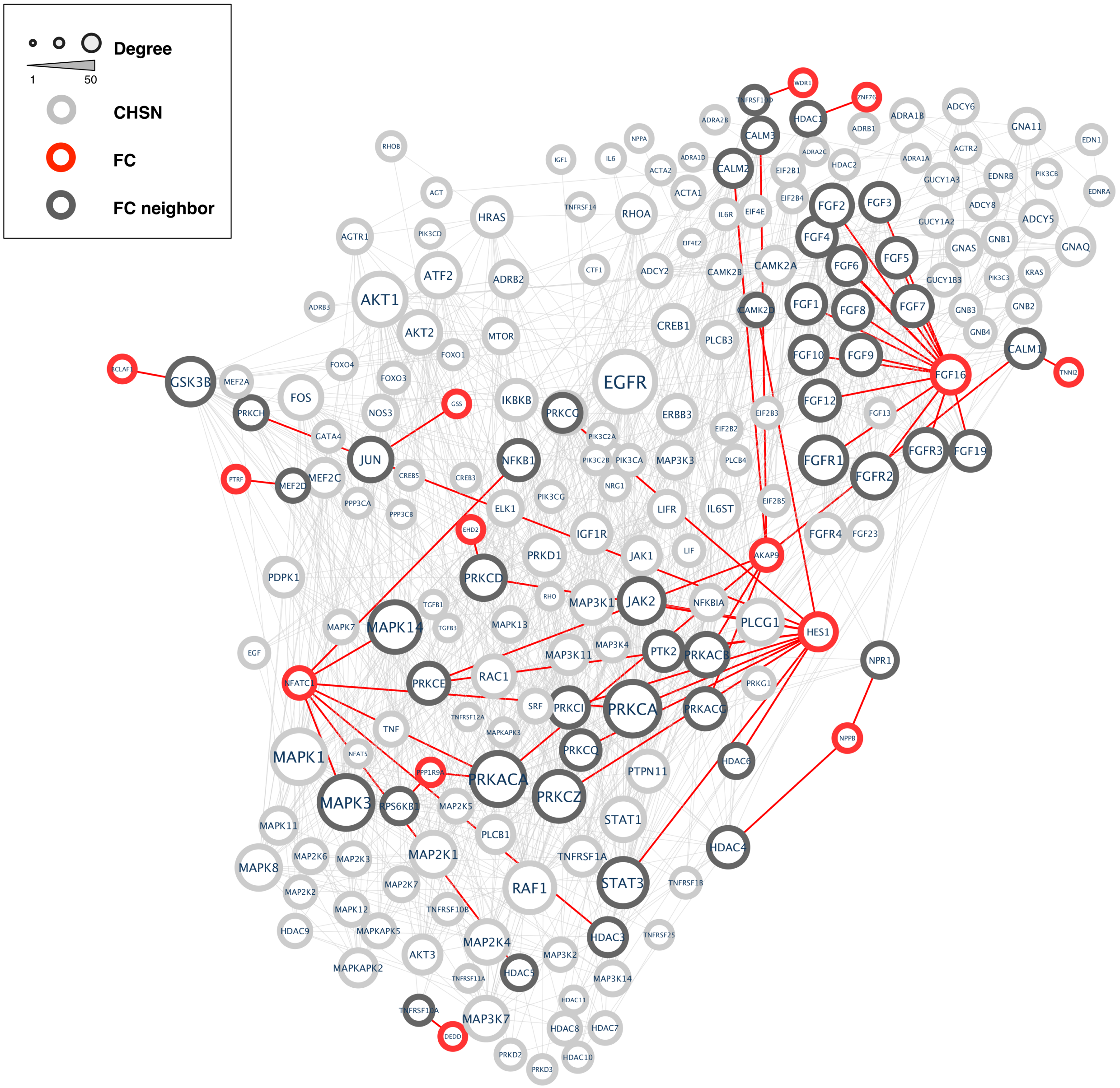

Figure S6

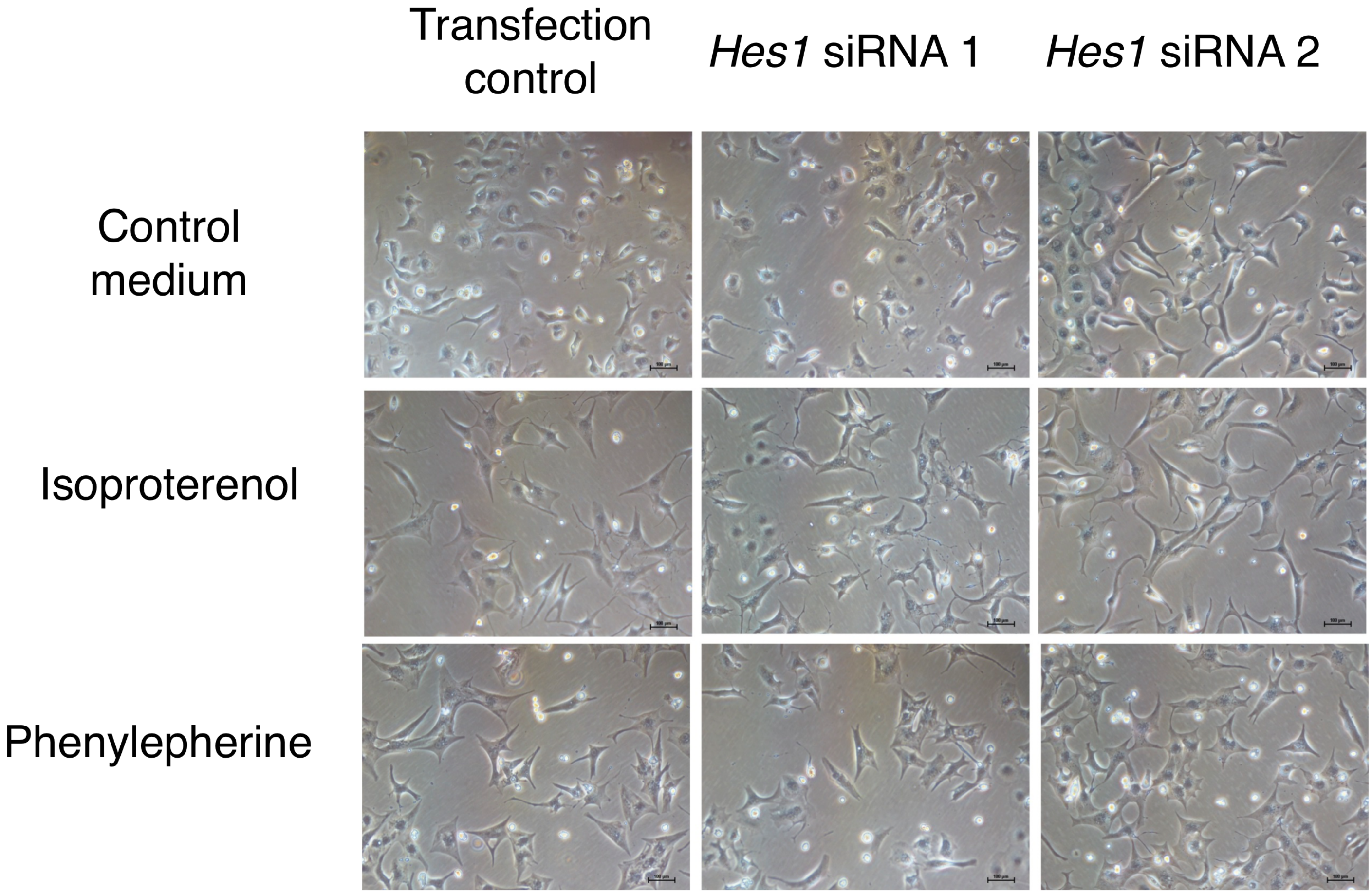

Transfection control
Hes1 siRNA 1
Hes1 siRNA 2
Control medium
Isoproterenol
Phenylepherine

Figure S7 *Hes1* **expression FC in the HMDP**

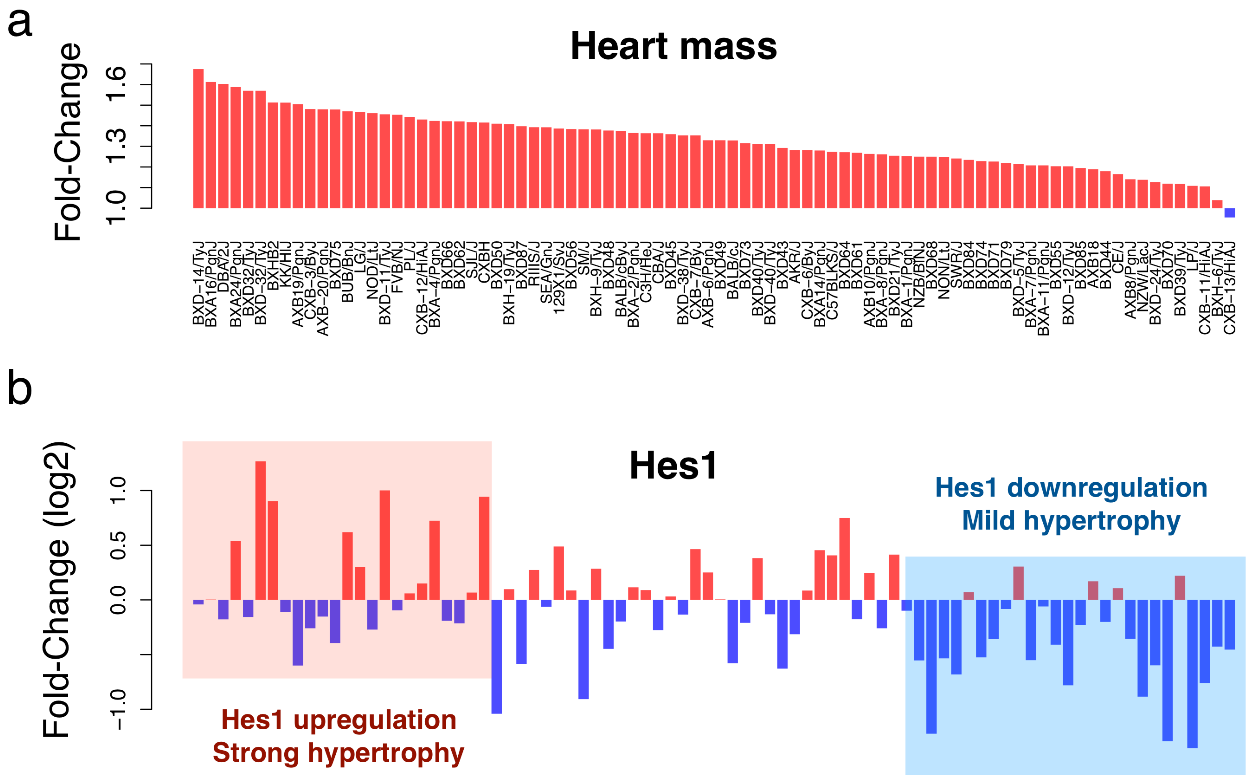

Supplement: Supplementary file 1 — Supplementary material [file 41540_2018_46_MOESM1_ESM.pdf]
